# Altered Gastrocnemius Contractile Behavior in Former Achilles Tendon Rupture Patients During Walking

**DOI:** 10.3389/fphys.2022.792576

**Published:** 2022-03-01

**Authors:** Benjamin Stäudle, Olivier Seynnes, Guido Laps, Gert-Peter Brüggemann, Kirsten Albracht

**Affiliations:** ^1^Faculty of Medical Engineering and Technomathematics, Aachen University of Applied Sciences, Aachen, Germany; ^2^Institute of Movement and Neurosciences, German Sport University Cologne, Cologne, Germany; ^3^Department of Physical Performance, Norwegian School of Sport Sciences, Oslo, Norway; ^4^Orthopädie am Gürzenich, Cologne, Germany; ^5^Institute of Biomechanics and Orthopedics, German Sport University Cologne, Cologne, Germany

**Keywords:** tendon rupture, muscle fascicle behavior, walking gait, force generation, ultrasound imaging

## Abstract

Achilles tendon rupture (ATR) remains associated with functional limitations years after injury. Architectural remodeling of the gastrocnemius medialis (GM) muscle is typically observed in the affected leg and may compensate force deficits caused by a longer tendon. Yet patients seem to retain functional limitations during—low-force—walking gait. To explore the potential limits imposed by the remodeled GM muscle-tendon unit (MTU) on walking gait, we examined the contractile behavior of muscle fascicles during the stance phase. In a cross-sectional design, we studied nine former patients (males; age: 45 ± 9 years; height: 180 ± 7 cm; weight: 83 ± 6 kg) with a history of complete unilateral ATR, approximately 4 years post-surgery. Using ultrasonography, GM tendon morphology, muscle architecture at rest, and fascicular behavior were assessed during walking at 1.5 m⋅s^–1^ on a treadmill. Walking patterns were recorded with a motion capture system. The unaffected leg served as control. Lower limbs kinematics were largely similar between legs during walking. Typical features of ATR-related MTU remodeling were observed during the stance sub-phases corresponding to series elastic element (SEE) lengthening (energy storage) and SEE shortening (energy release), with shorter GM fascicles (36 and 36%, respectively) and greater pennation angles (8° and 12°, respectively). However, relative to the optimal fascicle length for force production, fascicles operated at comparable length in both legs. Similarly, when expressed relative to optimal fascicle length, fascicle contraction velocity was not different between sides, except at the time-point of peak series elastic element (SEE) length, where it was 39 ± 49% lower in the affected leg. Concomitantly, fascicles rotation during contraction was greater in the affected leg during the whole stance-phase, and architectural gear ratios (AGR) was larger during SEE lengthening. Under the present testing conditions, former ATR patients had recovered a relatively symmetrical walking gait pattern. Differences in seen AGR seem to accommodate the profound changes in MTU architecture, limiting the required fascicle shortening velocity. Overall, the contractile behavior of the GM fascicles does not restrict length- or velocity-dependent force potentials during this locomotor task.

## Introduction

Recovery from Achilles tendon rupture (ATR) is a tedious process that results in permanent functional deficits in the majority of patients (Nilsson-Helander et al., 2010; Heikkinen et al., 2017). Such deficits are characterized by a weakness in end-range plantarflexion (Mullaney et al., 2006) and limited heel raise height (Silbernagel et al., 2012), and are often reflected by deficiencies during locomotor tasks (Willy et al., 2017). Gait deficiencies appear to be commensurate with movement velocity (Willy et al., 2017; Jandacka et al., 2018) and may therefore be related to the force-velocity relation of the affected muscles. Nonetheless, slow movements, — which require lower force levels — seem also impaired in most (Tengman and Riad, 2013; Agres et al., 2015; Willy et al., 2017; Speedtsberg et al., 2019) but not all investigated cases (Jandacka et al., 2017). The links between such deficits and the remodeling of the muscle-tendon unit (MTU) following ATR are, to date, poorly understood.

Long after recovery and regardless of treatment strategy, the MTU of ATR patients is characterized by a longer tendon (Silbernagel et al., 2012; Peng et al., 2019; Svensson et al., 2019). Recent studies suggest that the increased tendon stiffness (Agres et al., 2015; Stäudle et al., 2021) and shorter gastrocnemius medialis (GM) muscle fascicles (Baxter et al., 2018; Peng et al., 2019; Svensson et al., 2019) typically seen in ATR patients may compensate for their longer tendons, albeit incompletely (Stäudle et al., 2021). Using a musculoskeletal model to simulate maximum isometric contractions at various joint angles, we have shown that the shorter GM fascicles in the affected leg enables sarcomeres to operate close to their optimal length, but at the expense of a narrowed range for active force generation (Stäudle et al., 2021). The insights gained from these findings are, however, insufficient for predicting triceps surae mechanics in dynamic situations, where force-velocity conditions may set additional constraints.

As sarcomeres operate close to their optimal length during walking (Ishikawa et al., 2007), length-dependent deficits in muscle strength are expected to be rather small in ATR patients due to corresponding shorter fascicle lengths throughout the walking stance phase. Whereas, when considering the force-velocity relation of a muscle, shorter fascicles are expected to produce less force than longer ones at the same velocity because of their lower number of in-series sarcomeres (Hill, 1953; Bahler et al., 1968). This point may be critical in the case of ATR patients during walking, because of their shorter GM fascicle length and because of the force-limiting role of contractile velocity in the walking gait (Neptune and Sasaki, 2005; Farris and Sawicki, 2012). As the behavior of the remodeled muscle fascicles of ATR patients during walking gait has not yet been investigated and its impact on force generation is unclear, the purpose of this study was to investigate the hypothesis of altered contractile behavior of the GM causing a velocity-based deficit during walking in ATR patients. We expected the shorter GM fascicles of the affected leg to operate at a comparable length range but at a higher contractile velocity, relative to their optimal length, than the fascicles of the unaffected leg. Using combined ultrasonography and motion capture methods, we measured GM muscle mechanics in former ATR patients (more than 2 years post-surgery) walking at 1.5 m s^–1^.

## Materials and Methods

### Subjects

Male patients (20–60 years) were recruited for this study if they had suffered a complete ATR that had been treated surgically within 7 days after injury and were at least 2 years post-surgery. Subjects were excluded if they had a concomitant soleus muscle tear, sural nerve injury, or recurrent or bilateral ATR. The “Physical Activity Readiness Questionnaire” (Thomas et al., 1992) was used to exclude volunteers with cardiovascular or musculoskeletal disorders. The institutional review board of the German Sport University Cologne approved the study (approval number: 12/72), and all subjects provided written informed consent prior to voluntary participation.

This study is part of a comprehensive investigation on ATR patients’ functional deficits. For this purpose, sample size calculations were based on ATR patients’ strength deficits, as described previously (Stäudle et al., 2021). The *a priori* power analysis suggested a minimum sample size of 10 subjects.

Furthermore, GM tendon length, fascicle length, pennation angle and muscle thickness with a resting muscle had already been included in the previous study (Stäudle et al., 2021).

### Study Design and Experimental Protocol

**FIGURE 1:**
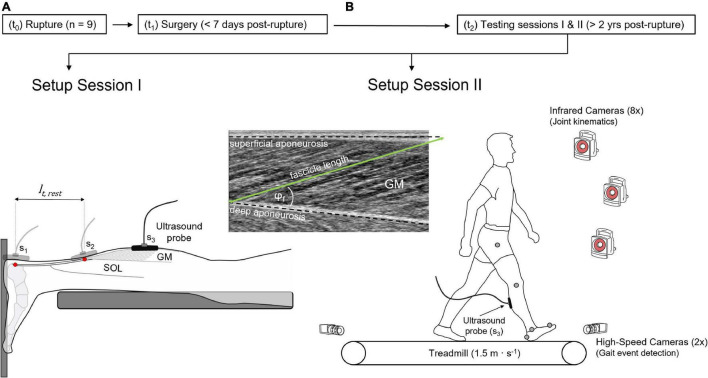
Experimental design. **(A)** Setup to determine gastrocnemius medialis (GM) tendon length and muscle architecture, showing the scanning location S_1_ and S_2_ of the ultrasound probe, visualizing the calcaneal insertion and the myotendinous junction of the GM tendon (red dots), respectively, defining GM tendon resting length (*l*_*t,rest*_). Scanning location 3 (S_3,_ GM mid-belly) visualizes GM muscle architecture in resting muscle **(A)** and during walking trials **(B)**, detailed by the ultrasound image. The treadmill setup **(B)** includes the motion capture system and the high-speed cameras. t_0_: time-point of rupture, t_1_: time-point of surgery, t_2_: time-point of measurements, φ_*f*_: pennation angle.

A cross-sectional design was used for this study. Data acquisition took place according to a pseudo randomized order between the affected and unaffected leg, while the unaffected leg served as control for matched comparison. During the first of two testing sessions (Figure 1), GM tendon length, fascicle length, pennation angle and muscle thickness were examined using ultrasonography with the subjects lying prone, with a resting muscle and ankle and knee joint angles in anatomical position (0°), as described previously (Stäudle et al., 2021). During the second session, subjects were familiarized to the treadmill by walking for about 5 min at 1.5 m⋅s^–1^ (h/p cosmos pulsar 4.0, 2.2 kW, Traunstein, Germany) using their own running shoes. Kinematic and ultrasonographic data were thereafter collected from each leg during six consecutive stance phases.

### Motion Capture of the Legs

A motion capture system (eight infrared cameras) sampling at 100 Hz (Vicon, Vicon Motion Systems Ltd., Oxford, United Kingdom) was used to capture knee and ankle joint kinematics. Anatomical landmarks were labeled *via* reflective markers representing the subjects’ greater trochanter, lateral femur condyle, lateral malleolus, calcaneus, and second metatarsal head. A static reference was captured with the ankle and knee joints in anatomical position to define 0°. For gait event detection (touch-down, toe-off), two additional high-speed cameras (Basler, 100 Hz, Ahrensburg, Germany) were positioned anteriorly and posteriorly to the treadmill belt.

### Measurements of Muscle Fascicle Behavior

During walking, B-mode ultrasound (Prosound α7, ALOKA, Tokyo, Japan) image sequences (73 Hz) were recorded using a t-shaped 6 cm linear array transducer (UST-5713T, 13 MHz) fixed in a custom-made cast to the mid-belly of the GM muscle *via* self-adhesive bandages. For time-synchronization, a rectangular voltage pulse was generated and sent to all data capturing devices.

Ultrasonography is a frequently used method to quantify muscle architecture under dynamic conditions (Cronin and Lichtwark, 2013) and shows good reliability within session for fascicle length and pennation angle in the present dataset (intraclass correlation coefficient: 0.99). In previous studies using similar methods, our group measured good inter-rater reliability with an intraclass correlation coefficient of 0.97 for pennation angle (Pohle-Fröhlich et al., 2020) and good reliability between days represented by a coefficient of multiple correlation of 0.93 for fascicle length and 0.87 for pennation angle (Aggeloussis et al., 2010).

A semi-automatic tracking algorithm (UltraTrack Software, version 4.2) was used to quantify GM muscle architecture. A dominant fascicle was drawn over a visible fascicle fragment and tracked across all frames. Additionally, superficial and deep aponeuroses were segmented and tracked. Fascicle length was defined as the distance between the insertions of the fascicles on the superficial and deep aponeuroses (Farris et al., 2016; Farris and Lichtwark, 2016).

In the rare exception where the transducer’s 6 cm width field of view failed to display the entire tracked fascicle (Figure 1), the missing portion was manually extrapolated, assuming that the fascicles and aponeurosis extend linearly. Linear extrapolation is associated with an error of less than 6% during maximal contractions of the GM muscle (Muramatsu et al., 2002). The pennation angle was defined as the angle between the muscle fascicle and the deep aponeurosis. Changes in series elastic element (SEE) length were estimated by subtracting muscle shortening amplitude from changes in MTU length. To this end, muscle shortening patterns were obtained from the length of the geometric projection of fascicles onto the axis of the deeper aponeurosis (Fukunaga et al., 2001). The length of the GM MTU was determined *via* a multiple linear regression equation using normative data based on joint angles and shank length (Hawkins and Hull, 1990).

### Data Processing

A custom-made script (MATLAB R2020b, The MathWorks, Inc., Natick, MA, United States) was used to analyze the data. Fascicle length and pennation angle were smoothed with a 5th order Butterworth lowpass filter at a 10 Hz cut-off frequency. Data were then time-normalized by being resampled to 101 data points and then averaged per stance. Muscle fascicle velocities were calculated as the time derivative of the respective lengths using the central difference method (Robertson et al., 2013). Marker trajectories of the kinematic measurement were smoothed with a Woltring filter (Generalized Cross Validation, smoothing: 10) (Vicon Nexus 2.2.2, Vicon Motion Systems Ltd., Oxford, United Kingdom).

All outcome parameters were obtained at the time-point of peak SEE length and calculated as average values during stance sub-phases of SEE lengthening and shortening. SEE lengthening was defined as the duration between initial ground contact and peak SEE length, the latter presumably indicating maximal SEE loading. SEE shortening was defined as the remaining duration to toe-off.

Kinematic parameters were also examined at additional time-points, to obtain an exhaustive characterization of potential changes in gait pattern. Thus, ankle and knee joint angles were analyzed at touch-down and toe-off, peak ankle joint dorsiflexion, and the first knee joint flexion angle local maximum. The range of motion of each joint was defined as the difference between the angles’ minima and maxima.

Variables that characterize fascicle behavior were also studied, including operating fascicle length, fascicle velocity, and pennation angle. In addition, fascicle and muscle shortening amplitudes, changes in pennation angle and architectural gear ratio (AGR) were analyzed for each of the two sub-phases. A modified version of the AGR (Brainerd and Azizi, 2005) was calculated as the ratio between muscle shortening amplitude along the axis of the deeper aponeurosis [calculated as the product of fascicle length by the cosine of the pennation angle (Fukunaga et al., 2001)] and fascicle shortening amplitude during the SEE sub-phases of walking (Werkhausen et al., 2019).

In addition to absolute values, the average operating fascicle length was expressed relative to optimal fascicle length and termed normalized operating fascicle length. Average fascicle velocity was expressed relative to the velocity of one optimal fascicle length per second and termed normalized fascicle velocity. Optimal fascicle length was estimated from resting fascicle length and normative data of sarcomere length using the following equation:


(1) $$l_{f,o}=\frac{l_{f,r\,e\,s\,t}}{l_{s,r\,e\,s\,t}}\cdot l_{s,o},$$

where *l*_*f,rest*_ is the fascicle length in resting condition (anatomically neutral position of the knee and ankle joint, 0°), *l*_*s,rest*_ the sarcomere length at the identical joint angles reported by Sanchez et al. (2015) (3.09 μm), and *l*_*s,o*_ the optimal sarcomere length of 2.725 μm defined by the mean value of the plateau region of the human sarcomere force-length relation (2.64–2.81 μm) (Herzog et al., 1990; Ward et al., 2009).

To illustrate the fraction of maximal GM force produced during walking, force potentials were estimated relative to operating length (length-dependent force potential) or velocity (velocity-dependent force potential). The length-dependent force potential was obtained using the default active-force-length curve of OpenSim based on quintic Bezier splines (Millard et al., 2013; Seth et al., 2018). As outlined in the OpenSim API guide (Millard, 2021), default parameters were chosen that the curve approximated the theoretical active-force-length curve of human sarcomeres (Nigg and Herzog, 1994) with the descending limb adapted from *in vitro* human fiber data (Gollapudi and Lin, 2009).

A dimensionless velocity-dependent force potential was obtained using the Hill-equation (Hill, 1938) for concentric contractions, relative values of force, velocity, and Hill’s constant *a* (Zajac, 1989) as follows:


(2) fv⁢(v′f)=(1-vf′v′m⁢a⁢x)1+(vf′v′m⁢a⁢x)⋅1ar⁢e⁢l,w⁢i⁢t⁢h⁢v′m⁢a⁢x=vm⁢a⁢x⋅(lf,o⋅s-1)-1.

*v_max_* is the GM muscle’s maximum shortening velocity, estimated as multiples of optimal fascicle length per second and considering the GM fraction of fast twitch fibers (Winters and Stark, 1988) as follows:


(3) $$v_{m\,a\,x}=\left(2+8\cdot F\,T\right)\cdot \left(l_{f,o}\cdot s^{-1}\right),$$

where the fraction of fast twitch (FT) fibers for the GM muscle was assumed to be 49.2% (Johnson et al., 1973). The normalized Hill constant *a*_*rel*_ was calculated from 0.1 + 0.4FT (Winters and Stark, 1988; Bohm et al., 2019), yielding 0.297.

### Statistical Analysis

Two tailed paired *t*-tests or Wilcoxon signed-rank tests were used to identify differences between the legs using Prism (version 7.04, GraphPad Software, Inc., San Diego, CA, United States). Prior to these tests, data were checked for normal distribution (Shapiro–Wilk normality test). Values are reported as means ± standard deviations (M ± SD) and the level of statistical significance was set to α ≤ 0.05. A statistical software package [G*Power version 3.1.9.6 (Faul et al., 2007)] was used to calculate absolute effect sizes, which were defined as small (0.2), moderate (0.5), or large (0.8), as appropriate (Cohen, 1988). An equivalent to effect sizes for signed rank tests was obtained by dividing the sum of the signed ranks by the total rank sum (matched pairs rank-biserial correlation) (Cureton, 1956; Kerby, 2014).

## Results

Out of 59 former ATR patients who responded to advertisements at a medical center (*n* = 45) or in media announcements (*n* = 14), 14 were recruited for this study. Of the remainder, 23 patients did not meet inclusion criteria, eight had moved, and 14 declined to participate. Nine of the 14 recruited subjects performed all tests and were included in the present data. The data from the five remaining subjects could not be obtained because of incomplete ultrasound scans (*n* = 2) or schedule conflicts (*n* = 3).

The subjects (age: 44.7 ± 9.1 years; height: 180 ± 7 cm; weight: 82.8 ± 5.9 kg) had been operated with either a modified Bunnel, a Kessler end-to-end or a Dresdner Instrument (minimal invasive) 3.4 ± 1.8 days after rupture and, 4.4 ± 2.1 years prior to the study, on average. Structural differences in muscles and tendons were observed in the affected legs, with shorter GM muscle fascicles [30.8 ± 10.8%, *t*(8) = 7.28, *p* < 0.001, *d*_*z*_ = 2.42], greater pennation angles [5 ± 3°, *t*(8) = 5.08, *p* = 0.001, *d*_*z*_ = 1.69], reduced muscle thickness [9.8 ± 12.4%, *t*(8) = 2.40, *p* = 0.043, *d*_*z*_ = 0.80], and longer GM tendons [13.9 ± 11.7%, *t*(8) = 3.73, *p* = 0.006, *d*_*z*_ = 1.25] (Supplementary Table 1).

### Kinematics

**FIGURE 2:**
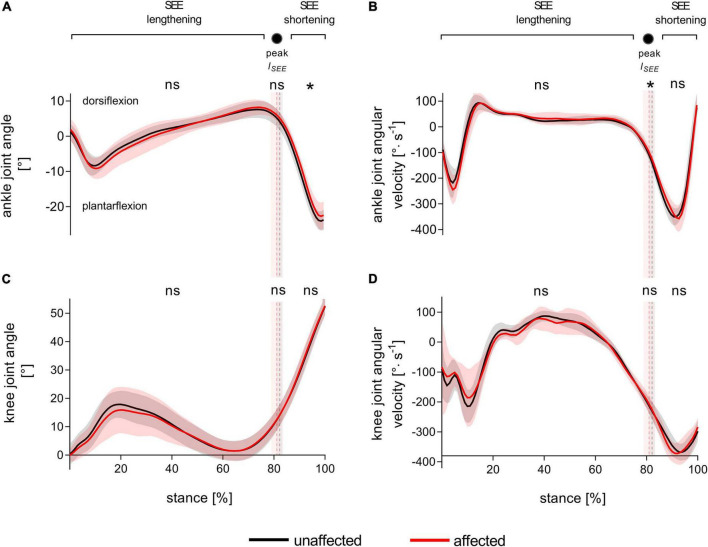
Ankle **(A)** and knee **(C)** joint angles and corresponding angular velocities **(B,D)** during walking stance. The solid black and red lines, with their corresponding shaded areas [standard deviation (SD)], represent mean traces for the unaffected and affected leg, respectively. The dashed vertical lines with their corresponding shaded areas (SD) represent the mean time points of peak series elastic element (SEE) length (peak *l*_*SEE*_) separating the SEE lengthening and shortening sub-phases. Statistical testing was run on average values during the SEE lengthening and shortening sub-phases, and on single data points at peak SEE length. *p < 0.05, significant side-to-side difference, ns: non-significant.

Figure 2 represents the knee and ankle joint angles and their corresponding angular velocities during stance. Except for a less plantarflexed averaged ankle joint angle during SEE shortening [3 ± 4°, *t*(8) = 2.34, *p* = 0.048, *d*_*z*_ = 0.78] and a slower ankle angular velocity at peak SEE length [21.5 ± 25.2%, *t*(8) = 2.41, *p* = 0.043, *d*_*z*_ = 0.80], the analyzed kinematic or spatio-temporal parameters did not show any significant differences (Figure 2, Table 1, and Supplementary Table 2). On average, the step frequency was 119 ± 5 steps⋅min^–1^.

**TABLE 1:** Leg kinematics and spatio-temporal parameters during walking stance.

Parameters		Affected		Unaffected		Diff		95% CI	Test statistic	*P*	Cohen’s d_*z*_
		*M*	SD	*M*	SD	*M*	SD				
Ankle joint	Range of motion (°)	32 (8)^w^		32 (6)^w^		0 (3)^w^		–	*W* = −9 (18, −27)	0.652	*r* = 0.20^w^
	Touch-down angle (°)	2 (4)^w^		2 (4)^w^		0 (5)^w^		–	*W* = 3 (24, −21)	0.910	*r* = 0.07^w^
	Toe off angle (°)	−22	4	−24	3	1	3	−1 to 4	*t*(8) = 1.22	0.256	0.41
	Max.-dorsiflexion (°)	8	2	8	2	1	3	−1 to 3	*t*(8) = 0.70	0.504	0.23
Knee joint	Range of motion (°)	54	6	54	7	0	5	−4 to 3	*t*(8) = 0.11	0.917	0.04
	Touch-down angle (°)	0	2	0	4	0	3	−3 to 2	*t*(8) = 0.36	0.732	0.12
	Toe-off angle (°)	53	3	52	3	0	3	−2 to 3	*t*(8) = 0.19	0.854	0.06
	Flexion (1st peak) (°)	16 (9)^w^		18 (8)^w^		0 (8)^w^		-	*W* = −9 (18, −27)	0.652	*r* = 0.20^w^
Stance duration	(s)	0.66	0.03	0.65	0.04	0.00	0.05	−0.03 to 0.04	*t*(8) = 0.14	0.890	0.05
Step length	(cm)	53.0 (6.0)^w^		52.0 (4.0)^w^		0 (1.5)^w^		–	*W* = 11 (13, −2)	0.250	*r* = 0.73^w^

*M, mean; SD, standard deviation; CI, confidence interval; ^w^, Wilcoxon signed rank test applied and values are expressed as median (interquartile range); W, sum of signed ranks (sum of positive, sum of negative ranks); r, matched pairs rank-biserial correlation.*

### Gastrocnemius Medialis Muscle Fascicle Length and Pennation Angle During Walking

The timing of maximum SEE elongation was used to separate the stance phase into SEE lengthening and shortening sub-phases. This time-point did not differ between the affected and unaffected leg [80.9 ± 2.2% vs. 82.3 ± 1.1%, *t*(8) = 1.76, *p* = 0.116, *d*_*z*_ = 0.59].

**FIGURE 3:**
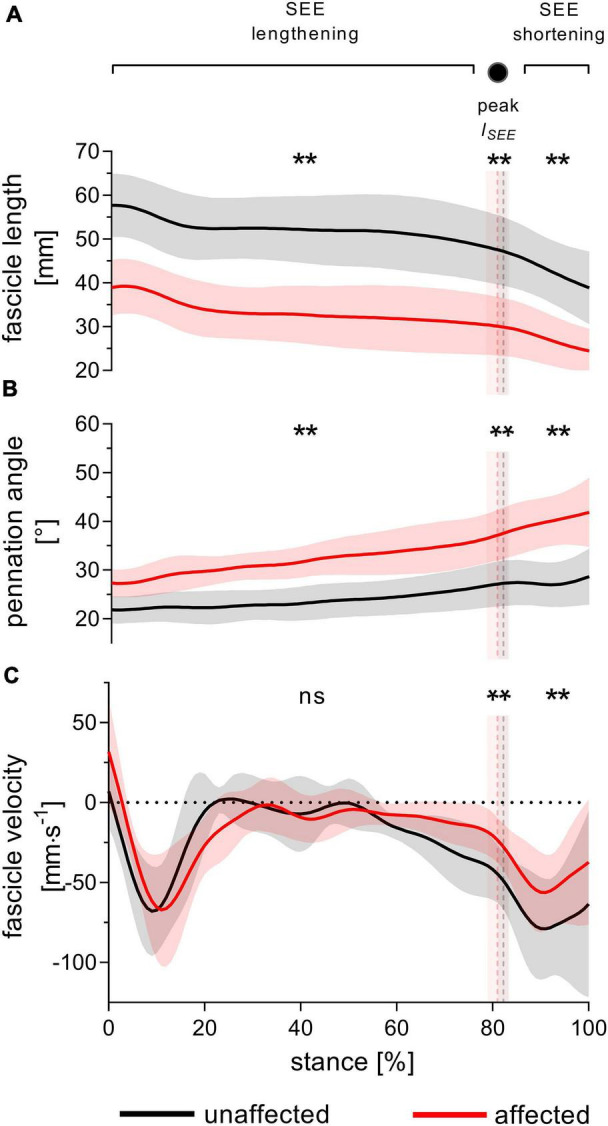
Gastrocnemius medialis operating fascicle length **(A)**, pennation angle **(B)** and fascicle velocity **(C)** during walking stance. The solid black and red lines, with their corresponding shaded areas [standard deviation (SD)], represent mean traces for the unaffected and affected leg, respectively. The dashed vertical lines with their corresponding shaded areas (SD) represent the mean time points of peak series elastic element (SEE) length (peak *l*_*SEE*_) separating the SEE lengthening and shortening sub-phases. Statistical testing was run on average values during SEE lengthening and shortening sub-phases, and on single data points at peak SEE length. **p < 0.01, significant side-to-side difference; ns: non-significant.

**FIGURE 4:**
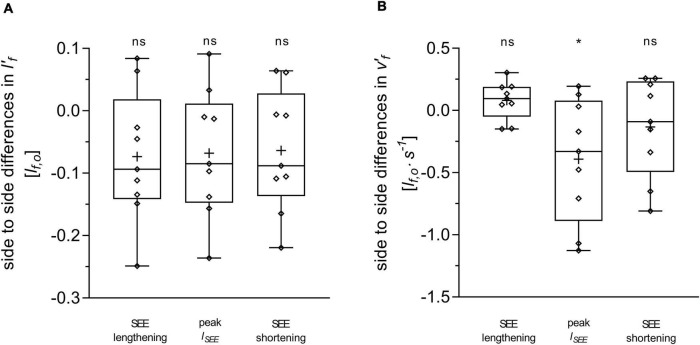
Differences between the affected and unaffected legs in gastrocnemius medialis normalized fascicle length $\left(l_{f}^{\prime }\right)$ **(A)** and normalized fascicle velocity $\left(v_{f}^{\prime }\right)$ **(B)**. Statistical testing was run on average values during the series elastic element (SEE) lengthening and shortening sub-phases, and on single data points at peak SEE length (*l*_*SEE*_). The lower and upper parts of the box plots represent the first and third quartile, respectively. The length of the whisker delineates the minimum and maximum values. The horizontal line in the box represents the median of the sample; +, sample mean; *p < 0.05, significant side-to-side difference; ns: non-significant.

**FIGURE 5:**
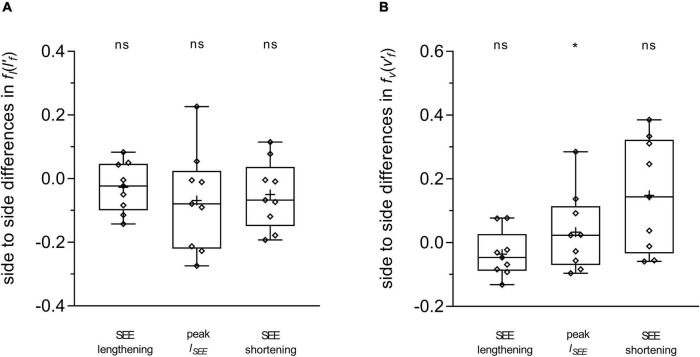
Differences between the affected and unaffected legs in gastrocnemius medialis length-dependent $\left[f_{l}\,\left(l_{f}^{\prime }\right)\right]$ **(A)** and velocity-dependent [$f_{v}\left(v_{f}^{\prime }\right)]$ **(B)** force potential. Statistical testing was run on average values during the series elastic element (SEE) lengthening and shortening sub-phases, and on single data points at peak SEE length (*l*_*SEE*_). The lower and upper parts of the box plots represent the first and third quartile, respectively. The length of the whisker delineates the minimum and maximum values. The horizontal line in the box represents the median of the sample; +, sample mean; *p < 0.05, significant side-to-side difference; ns: non-significant.

During SEE lengthening and shortening sub-phases, the average operating fascicle length was shorter in the affected leg (36.2 ± 8.5% and 36.3 ± 10.0%, respectively), which was consistent with a shorter operating fascicle length at peak SEE length in the same leg (36.2 ± 10.6%) (Table 2 and Figure 3A). Shortening amplitudes did not differ significantly during the lengthening sub-phase, whereas smaller shortening amplitudes were observed during the shortening sub-phase in the affected leg (26.0 ± 32.2%) (Table 3). However, after normalization to optimal length, the average operating fascicle length did not differ between legs in either of the stance sub-phases or peak SEE length (Table 2 and Figure 4A), nor did the average length-dependent force potential (Table 2 and Figure 5A).

**TABLE 2:** Architectural parameters of the gastrocnemius medialis muscle during walking stance.

Parameters	Stance sub-phase or time-point	Affected		Unaffected		Diff		95% CI	Test statistic	*P*	Cohen’s *d*_z_
		M	SD	M	SD	M	SD				
*l*_*f*_(*mm*)	SEE lengthening	33.3	6.6	52.2	7.7	−18.8	5.1	−22.7 to −14.9	*t*(8) = 11.10	< 0.001	3.71
	peak *l*_*SEE*_	30.0	6.6	47.0	7.5	−17.0	5.5	−21.2 to −12.7	*t*(8) = 9.27	< 0.001	3.10
	SEE shortening	27.4	5.9	43.0	7.3	−15.6	5.2	−19.6 to −11.6	*t*(8) = 8.93	< 0.001	2.98
*v*_*f*_ (mm⋅s^–1^)	SEE lengthening	−16.3	4.9	−19.6	2.5	3.3	5.6	−1 to 7.6	*t*(8) = 1.79	0.112	0.59
	peak *l*_*SEE*_	−22.4	17.3	−54.2	26.7	31.9	25.4	12.3 to 51.4	*t*(8) = 3.76	0.006	1.26
	SEE shortening	−44.2	18.4	−69.0	23.2	24.8	21.7	8.2 to 41.5	*t*(8) = 3.44	0.009	1.14
φ_*f*_ (°)	SEE lengthening	32	4	24	3	8	3	6 to 11	*t*(8) = 7.55	< 0.001	2.51
	peak *l*_*SEE*_	37	5	27	5	10	4	7 to 13	*t*(8) = 6.67	< 0.001	2.22
	SEE shortening	40	6	27	5	12	4	9 to 16	*t*(8) = 8.56	< 0.001	2.84
$l_{f}^{\prime }$ (*l*_*f,o*_)	SEE lengthening	0.88	0.09	0.95	0.12	−0.07	0.11	−0.15 to 0.01	*t*(8) = 2.10	0.069	0.70
	peak *l*_*SEE*_	0.79	0.10	0.86	0.11	−0.07	0.10	−0.15 to 0.01	*t*(8) = 1.99	0.081	0.67
	SEE shortening	0.72	0.09	0.79	0.11	−0.06	0.10	−0.14 to 0.01	*t*(8) = 1.95	0.087	0.65
$v_{f}^{\prime }$ (*l_*f,o*_ ⋅ s*^–^*^1^*)	SEE lengthening	0.44	0.15	0.36	0.06	0.08	0.15	−0.04 to 0.20	*t*(8) = 1.59	0.150	0.53
	peak *l*_*SEE*_	0.58	0.43	0.98	0.44	−0.39	0.49	−0.77 to −0.01	*t*(8) = 2.38	0.045	0.79
	SEE shortening	1.15	0.42	1.28	0.47	−0.13	0.40	−0.44 to 0.17	*t*(8) = 1.01	0.344	0.34
$f_{l}\,\left(l_{f}^{\prime }\right)$	SEE lengthening	0.93	0.06	0.96	0.07	−0.03	0.08	−0.09 to 0.03	*t*(8) = 1.03	0.332	0.34
	peak *l*_*SEE*_	0.85	0.11	0.90	0.15	−0.05	0.11	−0.13 to 0.03	*t*(8) = 1.41	0.195	0.47
	SEE shortening	0.75	0.14	0.82	0.21	−0.07	0.16	−0.19 to 0.05	*t*(8) = 1.31	0.228	0.44
$f_{v}\,\left(v_{f}^{\prime }\right)$	SEE lengthening	0.74	0.07	0.78	0.03	−0.04	0.07	−0.09 to 0.02	*t*(8) = 1.50	0.173	0.50
	peak *l*_*SEE*_	0.70	0.17	0.56	0.13	0.15	0.18	0.01 to 0.28	*t*(8) = 2.52	0.036	0.84
	SEE shortening	0.50	0.12	0.47	0.12	0.03	0.12	−0.06 to 0.13	*t*(8) = 0.81	0.439	0.27

*M, mean; SD, standard deviation; CI, confidence interval; SEE, series elastic element; peak l_SEE_, time-point of peak SEE length; l_f_, operating fascicle length; v_f_, fascicle velocity; φ_f_, pennation angle; $l_{f}^{\prime }$, normalized operating fascicle length; $v_{f}^{\prime }$, normalized fascicle velocity; $f_{l}\,\left(l_{f}^{\prime }\right)$, length-dependent force potential; $f_{v}\,\left(v_{f}^{\prime }\right)$, velocity-dependent force potential.*

**TABLE 3:** Changes in muscle architecture of the gastrocnemius medialis muscle during the series elastic element (SEE) lengthening and shortening sub-phases.

Parameters	Stance sub-phase	Affected		Unaffected		Diff		95% CI	Test statistic	*P*	Cohen’s d_*z*_
		M	SD	M	SD	M	SD				
Δ*l_*f*_* (mm)	SEE lengthening	8.9	2.7	10.7	1.3	−1.8	2.8	−4 to 0.4	*t*(8) = 1.93	0.090	0.64
	SEE shortening	5.6	2.0	8.1	2.2	−2.5	2.8	−4.7 to −0.4	*t*(8) = 2.68	0.028	0.89
Δφ*_*f*_* (°)	SEE lengthening	10	4	5	2	4	4	2 to 7	*t*(8) = 3.74	0.006	1.24
	SEE shortening	5	3	1	2	3	3	1 to 6	*t*(8) = 3.19	0.013	1.06
△*l_*m*_* (mm)	SEE lengthening	10.5	2.9	11.8	1.7	−1.3	3.3	−3.83 to 1.3	*t*(8) = 1.14	0.289	0.38
	SEE shortening	5.7	1.7	7.6	2.0	−1.9	2.7	−3.9 to 0.2	*t*(8) = 2.12	0.067	0.71
AGR	SEE lengthening	1.19	0.05	1.09	0.04	0.09	0.05	0.06 to 0.14	*t*(8) = 5.50	< 0.001	1.83
	SEE shortening	1.08	0.23	0.93	0.07	0.15	0.22	−0.02 to 0.32	*t*(8) = 1.96	0.085	0.66

*M, mean; SD, standard deviation; CI, confidence interval; △l_f_, fascicle shortening amplitude; Δφ_f_, change in pennation angle; △l_m_, muscle shortening amplitude (geometric fascicle projection); AGR, architectural gear ratio; AGR was calculated as the ratio between the muscle shortening amplitudes and fascicle shortening amplitudes.*

Average fascicle velocity did not differ between legs during the lengthening sub-phase, but differences were present during the SEE shortening phase (31.6 ± 28.3% lower in the affected leg, Table 2 and Figure 3C). To the time-point of peak SEE length, fascicle velocity was lower (56.5 ± 28.0%) (Table 2 and Figure 3C). No side-to-side differences were detected when velocities were expressed relative to optimal length in either sub-phase (Table 2 and Figure 4B), but being lower in the effected leg to the time-point of peak SEE length (39.2 ± 49.4%) (Table 2 and Figure 4B).

Likewise, the velocity-dependent force potential did not differ between legs during either sub-phase, while a greater velocity-dependent force potential was observed for the affected leg at the discrete time-point of peak SEE length (14.8 ± 17.7%) (Table 2 and Figure 5B).

The average pennation angle was greater in the affected leg during SEE lengthening and shortening (8 ± 3° and 12 ± 4°, respectively), which was consistent with a greater pennation angle at peak SEE length time-point in the same leg (10 ± 4°) (Table 2 and Figure 3B). Greater changes in pennation angle were also found in the affected leg during SEE lengthening (4 ± 4°) and shortening (3 ± 3°) sub-phases (Table 3). Representative ultrasound images of the affected and unaffected GM at different time-points during ground contact are presented in the Supplementary Figure 1.

Muscle shortening amplitude did not differ between the legs in either sub-phase, while the AGR was greater in the affected leg during the SEE lengthening (8.7 ± 4.9%), but not during the SEE shortening sub-phase (Table 3).

## Discussion

This study aimed to understand how the behavior of the MTU is altered in former ATR patients during walking, to accommodate the longer Achilles tendon and shorter GM fascicles caused by the injury. Despite the drastic changes in the MTU architecture of the affected leg, gross walking parameters were not found to be dissimilar between the legs. The GM fascicle behavior was different between the affected and unaffected legs, however, unfavorable contractile conditions for force production during stance were not observed. Relative to their shorter length, the operating length of the affected fascicles was preserved, and the shortening velocity did not differ or was actually lower than in the healthy leg at the time of peak force production (as estimated from the timing of peak SEE length). These results indicate that under the tested walking conditions, the MTU recovery in former ATR patients may be sufficient to enable symmetrical walking gait. In addition, they suggest that long after recovery, GM fascicle behavior does not limit force generation potential and that remaining functional deficits during walking are attributable to other factors.

### Leg Kinematics During Walking

The similar ankle and knee joint kinematics between the affected and unaffected legs indicate that the ATR patients had recovered a symmetrical gait pattern during treadmill walking. Two exceptions were found with a less plantarflexed average ankle joint angle during the push-off sub-phase and a lower angular velocity of that joint in the affected leg at the instant of peak SEE length. Since no side-to-side difference in angular excursion was detected during SEE shortening, the functional significance of a more dorsiflexed angle during the push-off of the affected leg is elusive. These differences may be congruent with several, but not all (Jandacka et al., 2018), reports documenting small kinematic differences in patients with similar characteristics. Several authors investigating leg kinematics during overground walking found greater dorsiflexion angles in the affected leg than in the unaffected leg at touch-down (∼1°) (Tengman and Riad, 2013; Speedtsberg et al., 2019), at toe-off (∼4°) (Willy et al., 2017), and at peak joint flexion (∼1–4°) (Don et al., 2007; Tengman and Riad, 2013; Agres et al., 2015; Speedtsberg et al., 2019). Such alterations in ankle joint kinematics, particularly greater dorsiflexion angles, could be related to the lengthened tendon caused by ATR (Agres et al., 2015). For this reason, side-to-side differences in joint kinematics may be proportional to the degree of MTU remodeling—and slackness reduction—characterizing individual recovery. Of note, the contrasting experimental modalities (walking on a treadmill or overground) between studies may have set slight differences in gait requirements, arguably less challenging in the present study. Walking on a treadmill seems indeed to reduce ground reaction forces compared to walking overground (Riley et al., 2007; Parvataneni et al., 2009), which may have lowered the GM force requirements for our patients. Regardless, the present results suggest that the remaining side-to-side differences in kinematics are small and that the gross walking pattern is relatively symmetrical. They also suggest that any difference in fascicle behavior is likely not related to dissimilarities in joint configuration.

### The Gastrocnemius Medialis Fascicle Behavior Does Not Seem to Limit Force Production During Walking Stance

In support of our hypothesis, the operating GM fascicle length of the affected leg did not seem to limit potential force production. If fascicle length normalized to optimal length reflects sarcomere length, the sarcomeres of the affected leg appeared to work in a region of their force-length relation similar to that in the unaffected leg. These observations reinforce the notion that fascicle remodeling in ATR patients is achieved *via* a reduction in sarcomeres in series, as previously shown in animal studies on muscles immobilized at short lengths (Williams and Goldspink, 1973).

Unlike their operating length, we expected the fascicles of the affected leg to operate in an unfavorable region of their force-velocity relationship, explaining some of the functional limitations previously observed during walking. In fact, our data demonstrate that for most of the stance phase, fascicles do not operate at different normalized velocities between the legs. Conversely, affected fascicles operate in a more favorable region of their force-velocity relationship at peak SEE length, which is supported by a 15 ± 18% higher velocity-dependent force potential.

The observation of a slower contractile velocity also seems consistent with the slower ankle joint angular velocity at the time of peak SEE length. Such a reduction was likely not caused by an altered amount of fascicle shortening, since we did not find any difference in this parameter during the lengthening sub-phase. Therefore, it is difficult to elucidate the mechanisms responsible for the unchanged or reduced velocity from the present data.

One architectural factor may explain how the shorter fascicles of the affected leg could avoid contracting at a higher velocity than in the healthy leg: their rotation about their insertion point (i.e., change in pennation angle). This parameter being larger (4 ± 4°) in the affected leg may have limited the required fascicle contraction amplitude for a given muscle shortening, thereby providing a greater AGR. The present data support this interpretation, with a 9 ± 5% greater AGR in the affected leg during SEE lengthening. Thus, the contribution of the fascicle rotation to total muscle shortening during the SEE lengthening sub-phase accounted 16 ± 4% (1.6 ± 0.4 mm) on the affected leg and just 9 ± 4% (1.0 ± 0.5 mm) in the unaffected leg.

Variable AGR in general provides a mechanism to modulate performance during mechanically diverse functions (Azizi et al., 2008). The greater pennation angle after MTU remodeling of ATR patients may also promote fascicle rotation (Brainerd and Azizi, 2005), supporting architectural gearing. Architectural gearing may, therefore, constitute another adaptive mechanism in ATR patients, allowing maintaining the required muscle shortening velocity with a reduced fascicle velocity.

In the same line of thought, the altered mechanical properties of the GM tendon observed in ATR patients (Agres et al., 2015; Geremia et al., 2015; Stäudle et al., 2021) may also affect the contractile behavior of the muscle fascicles, *via* the effect of in series compliance on fascicle operating length and contraction velocity. We hypothesize that an increased stiffness counteracts the larger strain expected in the longer tendons of the affected side. This trade-off may thus limit the shortening of muscle fibers, while more compliant tendons would promote the opposite behavior. Since fascicular velocity was maintained or decreased on the affected side despite longer tendons, tendon stiffness was likely higher in the present patients, which could represent another adaptive mechanism in ATR patients to maintain function.

### Functional Implications

The present findings indicate that affected GM sarcomeres still operate within a favorable length range and at favorable velocities for force production during treadmill walking. This is particularly interesting, as, contrary to our hypothesis, fascicle behavior does not appear to explain the functional limitations observed in ATR patients during walking (Tengman and Riad, 2013; Willy et al., 2017; Speedtsberg et al., 2019).

However, the question remains whether ATR patients can fully regain the plantar flexion force required for walking. Substantial atrophy (Heikkinen et al., 2017), as shown here by a 10 ± 12% reduction in GM thickness, is usually measured in the transversal plane of the affected fascicles, reflecting a reduced capacity for maximal force production compared to the unaffected leg. In the present experiment, the remodeled—smaller—GM muscle may have produced marginally less force during most of the stance phase. However, we contend that the force requirements of our walking experimental conditions were sufficiently low and met by compensatory mechanisms without substantially affecting gait symmetry. Although this study was not designed to investigate such mechanisms, an increase in muscle activation such as that previously reported (Wenning et al., 2021) likely occurred here.

It follows that locomotor deficits may be force- and gait-dependent in ATR patients. This hypothesis is consistent with the data of Jandacka et al. (2018), who did not find functional deficit in ATR patients during overground walking, but observed such deficits in the affected leg during running. Future studies should systematically investigate the effects of gait velocity and ground reaction forces on fascicle behavior and force production capacity to describe muscle function in a more comprehensive manner.

### Limitations

A few aspects deserve further consideration for the interpretation of the present data. The final sample size (*n* = 9) was lower than the sample size (*n* ≥ 10) suggested after our *a priori* power analysis. Dropouts and exclusions of data of insufficient quality could not be prevented. However, the effect sizes of the differences discussed here are satisfactory; nevertheless, for completeness, the results should be verified in a future study against a matched control population with a greater sample size.

Furthermore, we used mean values of variables describing fascicular behavior. Although this approach omits testing differences at discrete time points, this choice was justified by two criteria. Firstly, it was our intention to capture substantial differences relative to tissue loading and unloading. Secondly, there was no obvious rationale to support the choice of discrete time points. Additionally, statistical parametric mapping analyses done *a posteriori* (Supplementary Figure 2; Bankosz and Winiarski, 2020) did not suggest that significant differences may have been masked by the present approach.

In general, the length- and velocity-dependent force potentials are based on the assumptions of biological consistency and best-fit approximations. Consequently, no model is exempt from inaccuracies. Our models could have been enhanced with additional data collection as previously described for the force-length relationship (de Brito Fontana and Herzog, 2016; Nikolaidou et al., 2017; Bohm et al., 2019, 2021) and the force-velocity relationship (Hauraix et al., 2015). Unfortunately, the large project encompassing this study did not allow additional measurements.

Similarly, for the calculations of the velocity-dependent force potential, maximum shortening velocity and normalized Hill constant (*a*_*rel*_) were estimated based on normative data for fiber type distribution in GM muscles (type I fibers: 50.8%, type II fibers: 49.2%) (Johnson et al., 1973). As we cannot discard the possible influence of this parameter on side-to-side differences, speculating about the potential role of fiber type distribution in the present data should be avoided.

## Conclusion

The results of this study indicate that sarcomere operating length and lower limbs kinematics are relatively preserved during walking in ATR patients more than 2 years post-surgery. Despite atrophy in the longitudinal and transversal planes of the affected GM muscle, fascicles were found to operate at contractile velocities comparable to or lower than those of the non-operated leg during the stance phase. An increased fascicle rotation during stance may contribute to preserving fascicle contractile velocity. Collectively, these results suggest that the contractile behavior of the GM muscle does not limit force production under the present walking condition and in ATR patients with a similar level of recovery.

## Data Availability Statement

The raw data supporting the conclusions of this article will be made available by the authors, without undue reservation.

## Ethics Statement

The studies involving human participants were reviewed and approved by the institutional review board of the German Sport University Cologne. The patients/participants provided their written informed consent to participate in this study.

## Author Contributions

G-PB and KA conceptualized the research. BS, GL, G-PB, and KA designed the research. BS acquired the data. BS and KA analyzed the data. BS, OS, and KA interpreted the data and drafted the manuscript. BS, OS, GL, G-PB, and KA revised the manuscript. All authors approved the final manuscript.

## Conflict of Interest

The authors declare that the research was conducted in the absence of any commercial or financial relationships that could be construed as a potential conflict of interest.

## Publisher’s Note

All claims expressed in this article are solely those of the authors and do not necessarily represent those of their affiliated organizations, or those of the publisher, the editors and the reviewers. Any product that may be evaluated in this article, or claim that may be made by its manufacturer, is not guaranteed or endorsed by the publisher.

## Abbreviations

AGR: architectural gear ratio
ATR: Achilles tendon rupture
FT: fast twitch
ICC: intra-class correlation coefficient
GM: gastrocnemius medialis
MTU: muscle-tendon unit
SEE: series elastic element.
