# Supplementary material for: Altered Gastrocnemius Contractile Behavior in Former Achilles Tendon Rupture Patients During Walking
Source: Front Physiol. 2022 Mar 1;13:792576. doi: 10.3389/fphys.2022.792576 (PMC8921480; doi:10.3389/fphys.2022.792576)
Supplement: Supplementary file 1 [file Data_Sheet_1.pdf]

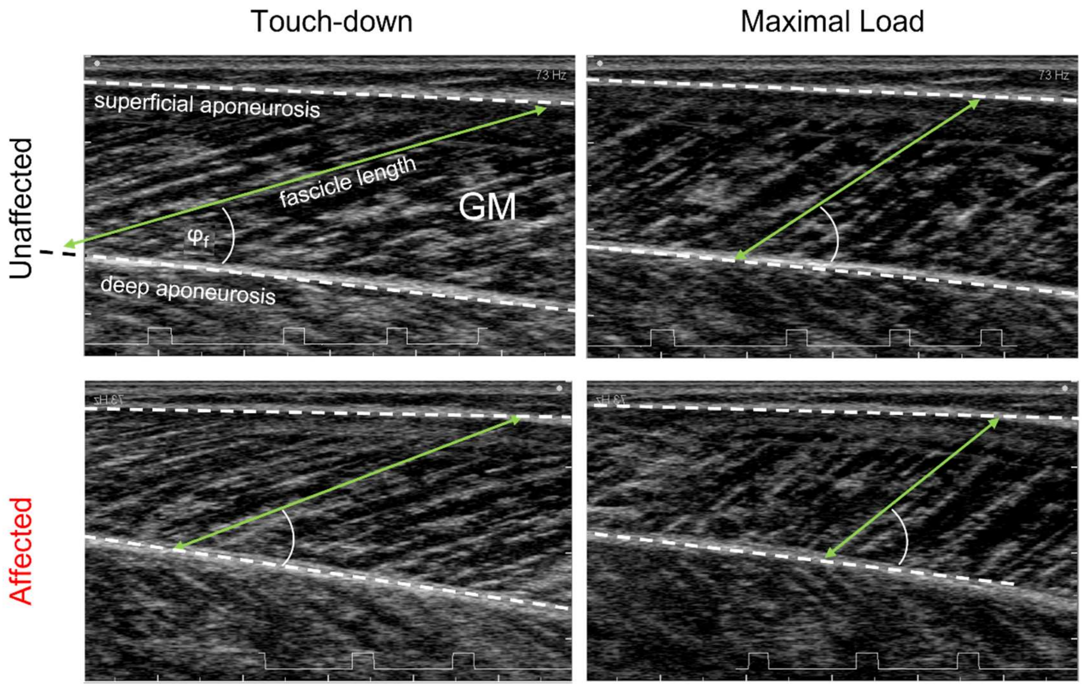

Supplementary Figure 1 Ultrasound images of the gastrocnemius medialis (GM) muscle. The unaffected (upper images) and affected (lower images) GM muscles are represented at touch-down (initial foot contact) and to maximal load (peak series elastic element length) of the gait cycle. Compared to the unaffected side, affected fascicles show shorter fascicle length and greater pennation angles during walking stance. φ_f: pennation angle.
