# Supplementary material for: Altered Gastrocnemius Contractile Behavior in Former Achilles Tendon Rupture Patients During Walking
Source: Front Physiol. 2022 Mar 1;13:792576. doi: 10.3389/fphys.2022.792576 (PMC8921480; doi:10.3389/fphys.2022.792576)
Supplement: Supplementary file 2 [file Data_Sheet_2.pdf]

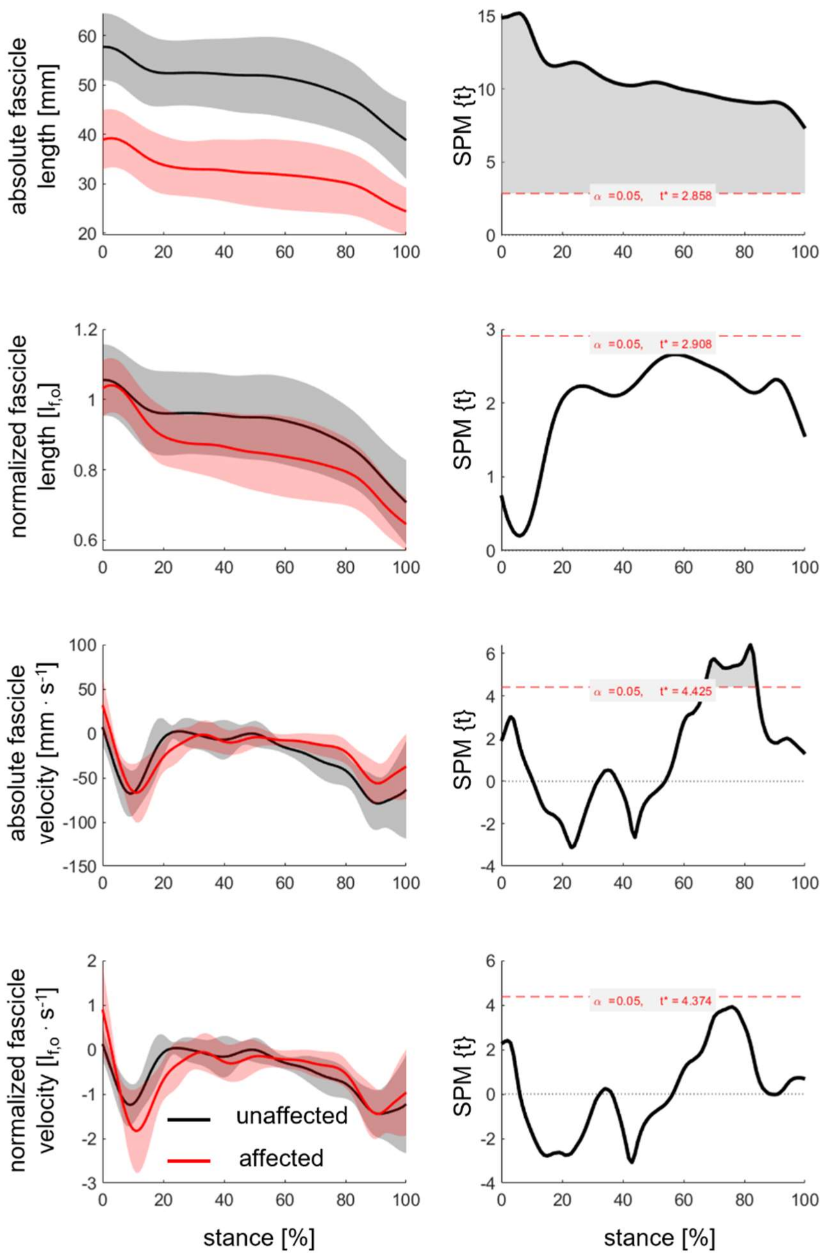

Supplementary Figure 1 The statistical parametric mapping (SPM) for absolute and normalized fascicle length and velocity. Figures to the left side represent mean traces (solid line) and corresponding standard deviation (shaded area) for the unaffected (black) and affected (red) leg. Figures to the right side represent the corresponding SPM analysis. SPM was calculated with a two tailed paired t-test with α = 0.05. The grey area underneath the SPM {t} - curve indicates significant differences between the unaffected and affected side. t*: test statistic.
