# Supplementary material for: Altered Gastrocnemius Contractile Behavior in Former Achilles Tendon Rupture Patients During Walking
Source: Front Physiol. 2022 Mar 1;13:792576. doi: 10.3389/fphys.2022.792576 (PMC8921480; doi:10.3389/fphys.2022.792576)
Supplement: Supplementary file 3 [file Table_1.pdf]

Supplementary Table 1 GM muscle architecture and tendon morphology at rest

Parameters	Affected		Unaffected		Diff		95% CI	Test statistic	P	Cohen's d_z
	M	SD	M	SD	M	SD				
$l_{t,rest}$ (mm)	227	12	200	15	26	21	10 to 43	t(8) = 3.73	.006	1.25
$l_{f,rest}$ (mm)	42.7	6.1	62.1	6.4	-19.4	8.0	-25.6 to -13.3	t(8) = 7.28	<.001	2.42
$l_{f,o}$ (mm)	37.7	5.3	54.8	5.6	-17.1	7.1	-22.6 to -11.7	t(8) = 7.28	<.001	2.42
$\varphi_{f,rest}$ (°)	25	3	20	2	5	3	3 to 8	t(8) = 5.08	.001	1.69
$t_{m,rest}$ (mm)	17.0	2.8	18.8	1.4	-1.8	2.3	-3.6 to -0.1	t(8) = 2.40	.043	0.80

M, mean; SD, standard deviation; CI, confidence interval; GM, gastrocnemius medialis muscle; $l_{t,rest}$, tendon resting length; $l_{f,rest}$, fascicle resting length; $l_{f,o}$, optimal fascicle length for force production; $\varphi_{f,rest}$, pennation angle at rest; $t_{m,rest}$, muscle resting thickness.
