# Supplementary material for: Altered Gastrocnemius Contractile Behavior in Former Achilles Tendon Rupture Patients During Walking
Source: Front Physiol. 2022 Mar 1;13:792576. doi: 10.3389/fphys.2022.792576 (PMC8921480; doi:10.3389/fphys.2022.792576)
Supplement: Supplementary file 4 [file Table_2.pdf]

Supplementary Table 2 Kinematic parameters averaged over series elastic element (SEE) sub-phases and to the time-point of peak SEE length (l_{SEE})

		Affected		Unaffected		Diff		95% CI	Test statistic	P	Cohen's d_z
Parameters	Stance sub-phase or time-point	M	SD	M	SD	M	SD				
Ankle joint angle (°)	SEE lengthening	1	2	2	2	0	2	-2 to 1	t(8) = 0.52	.620	0.17
	peak l_{SEE}	6	3	5	3	2	3	0 to 4	t(8) = 1.84	.103	0.61
	SEE shortening	-8	2	-11	3	3	4	0 to 6	t(8) = 2.34	.048	0.78
Ankle joint angular velocity (°·s^{-1})	SEE lengthening	7.25	6.20	4.96	4.05	2.29	4.94	-1.50 to 6.09	t(8) = 1.39	.201	0.46
	peak l_{SEE}	-102	31	-136	37	34	43	1 to 67	t(8) = 2.41	.043	0.80
	SEE shortening	-212	20	-225	27	13	19	-2 to 28	t(8) = 2.03	.077	0.68
Knee joint angle (°)	SEE lengthening	8	5	9	4	-1	3	-3 to 2	t(8) = 0.63	.545	0.21
	peak l_{SEE}	13	3	14	3	-1	5	-5 to 2	t(8) = 0.90	.397	0.30
	SEE shortening	32	4	32	3	-1	4	-4 to 3	t(8) = 0.55	.595	0.18
Knee joint angular velocity (°·s^{-1})	SEE lengthening	-25.1	7.6	-26.3	6.1	1.2	8.9	-5.6 to 8.1	t(8) = 0.41	.694	0.14
	peak l_{SEE}	-210	57	231	32	21	52	-19 to 60	t(8) = 1.19	.267	0.40
	SEE shortening	-315	40	-320	26	5	30	-17 to 28	t(8) = 0.55	.600	0.18

M, mean; SD, standard deviation; CI, confidence interval.
